# Cæsarean Section Successfully Performed; Both Mother and Child Saved

**Published:** 1844-02-10

**Authors:** 


CÆSAREAN SECTION SUCCESSFULLY PERFORMED; BOTH MOTHER AND CHILD SAVED.

A woman, aged thirty-one, who had borne five children naturally, was attacked with violent arthritis during her sixth pregnancy. The pelvis became so deformed that the finger could scarcely be introduced between the tuberosities of the ischium and the ascending rami, on either side; the pubes also formed a very prominent angle, the sacrum projected much forwards, and the os uteri could not be reached. On the 27th of July, 1840, labour having commenced, and the contraction of the pelvic diameter being well ascertained, the Cæsarean section was determined on, and was performed in the linea alba by Dr. Arnoldi. The results were most fortunate; the mother nursed the child herself, and the wound healed by the beginning of September.—*Ibid, from Casper's Wochenschrift.*

